# Systems Pharmacology and Rational Polypharmacy: Nitric Oxide−Cyclic GMP Signaling Pathway as an Illustrative Example and Derivation of the General Case

**DOI:** 10.1371/journal.pcbi.1004822

**Published:** 2016-03-17

**Authors:** Farshid S. Garmaroudi, Diane E. Handy, Yang-Yu Liu, Joseph Loscalzo

**Affiliations:** 1 Cardiovascular Division, Department of Medicine, Brigham and Women's Hospital and Harvard Medical School, Boston, Massachusetts, United States of America; 2 Channing Division of Network Medicine, Department of Medicine, Brigham and Women's Hospital and Harvard Medical School, Boston, Massachusetts, United States of America; University of Southern California, UNITED STATES

## Abstract

Impaired nitric oxide (NO˙)-cyclic guanosine 3', 5'-monophosphate (cGMP) signaling has been observed in many cardiovascular disorders, including heart failure and pulmonary arterial hypertension. There are several enzymatic determinants of cGMP levels in this pathway, including soluble guanylyl cyclase (sGC) itself, the NO˙-activated form of sGC, and phosphodiesterase(s) (PDE). Therapies for some of these disorders with PDE inhibitors have been successful at increasing cGMP levels in both cardiac and vascular tissues. However, at the systems level, it is not clear whether perturbation of PDE alone, under oxidative stress, is the best approach for increasing cGMP levels as compared with perturbation of other potential pathway targets, either alone or in combination. Here, we develop a model-based approach to perturbing this pathway, focusing on single reactions, pairs of reactions, or trios of reactions as targets, then monitoring the theoretical effects of these interventions on cGMP levels. Single perturbations of all reaction steps within this pathway demonstrated that three reaction steps, including the oxidation of sGC, NO˙ dissociation from sGC, and cGMP degradation by PDE, exerted a dominant influence on cGMP accumulation relative to other reaction steps. Furthermore, among all possible single, paired, and triple perturbations of this pathway, the combined perturbations of these three reaction steps had the greatest impact on cGMP accumulation. These computational findings were confirmed in cell-based experiments. We conclude that a combined perturbation of the oxidatively-impaired NO˙-cGMP signaling pathway is a better approach to the restoration of cGMP levels as compared with corresponding individual perturbations. This approach may also yield improved therapeutic responses in other complex pharmacologically amenable pathways.

## Author Summary

Developing drugs for a well-defined biochemical or molecular pathway has conventionally been approached by optimizing the inhibition (or activation) of a single target by a single pharmacologic agent. On occasion, drug combinations have been used that generally target multiple pathways affecting a common phenotype, again by optimizing the extent of inhibition of individual targets, semi-empirically adjusting their doses to minimize toxicities as they are manifest. Here, we present a computational approach for identifying optimal combinations of agents that can affect (inhibit) a well-defined biochemical pathway, doing so at minimal combined concentrations, thereby potentially minimizing dose-dependent toxicities. This approach is illustrated computationally and experimentally with a well-known pathway, the nitric oxide-cyclic GMP pathway, but is readily generalizable to rational polypharmacy.

## Introduction

**Fig 1:**
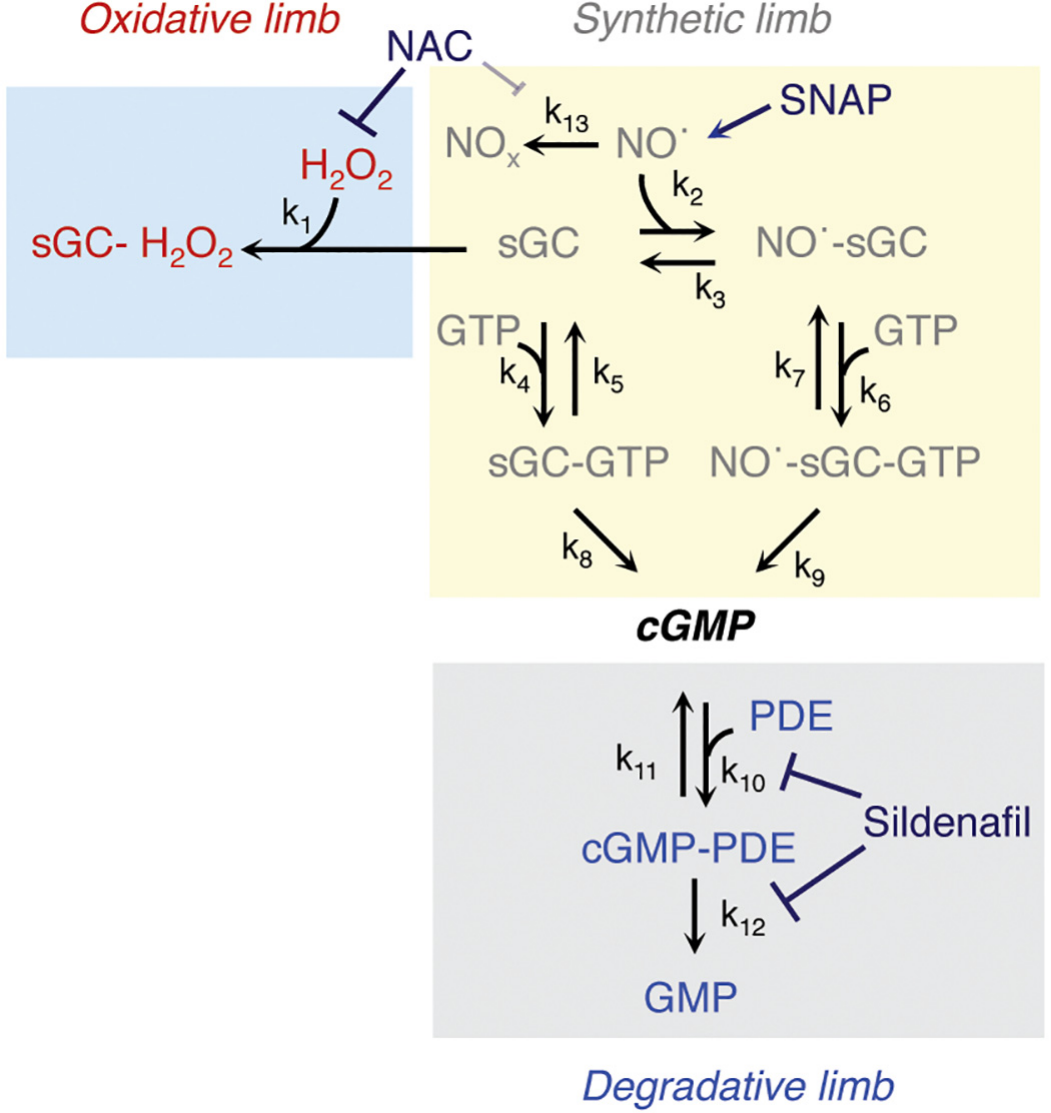
Steps for modeling NO˙-cGMP signaling pathway. The reaction schema: NO˙ is synthesized in a generator cell and freely diffuses, either within the same cell or to a target cell, to activate sGC; sGC is oxidized by H_2_O_2_ and inactivated. Either sGC or NO˙-sGC can convert GTP to cGMP, which is degraded by PDE to GMP. Note that oxidative stress drives the system toward the oxidative limb, and that the goal of pharmacological modulation of this pathway is to reverse the adverse effects of oxidative stress and to minimize PDE inhibition in order to optimize cGMP levels. NAC can be used as an antioxidant (impairing hydrogen peroxide-dependent oxidation of SGC and, perhaps, oxidation of NO˙), sildenafil as a PDE5 inhibitor, and SNAP as an NO˙ donor to modulate this pathway experimentally. Abbreviations: cGMP, cyclic guanosine 3', 5'-monophosphate; GMP, guanosine-5'-monophosphate; GTP, guanosine-5'-triphosphate; H_2_O_2_, hydrogen peroxide; NAC, N-acetylcysteine; NO˙, nitric oxide; NO_x_, oxidized (inactive) nitrogen oxides; PDE, phosphodiesterase; SNAP, S-Nitroso-n-nacetylpenicillamine; sGC, soluble guanylyl cyclase.

Signal transduction via the nitric oxide (NO˙)-cyclic guanosine 3', 5'-monophosphate (cGMP) pathway is involved in multiple and diverse biological responses, including smooth muscle relaxation, inhibition of platelet aggregation, and neural communication [1–6]. This pathway is composed of several molecular species acting in two opposing limbs, the cGMP-synthetic limb and the cGMP-degradative limb (see Fig 1). The proper function of these two limbs is crucial in controlling these biological responses. Within the cGMP-synthetic limb, NO˙ binds to soluble guanylyl cyclase (sGC) to catalyze the production of cGMP from guanosine-5'-triphosphate (GTP), whereas in the cGMP-degradative limb, cyclic nucleotide phosphodiesterase (PDE) converts cGMP to GMP. Impaired function of either or both limbs of the NO˙-cGMP signaling pathway has been reported in many cardiovascular disorders, including heart failure and pulmonary arterial hypertension.

Importantly, increased oxidative stress associated with malfunction of the NO˙-cGMP signaling pathway has been implicated in the pathobiology of several diseases [7, 8]. During oxidative stress, the pathway’s unresponsiveness can be explained by several mechanisms, among which sGC insensitivity to NO˙ (tolerance) is decisive. Elevated reactive oxygen species (ROS) may promote sGC insensitivity through either non-heme (cysteine) oxidation of sGC [9–14], S-nitrosation of sGC [15], heme oxidation of sGC [16], or oxidation of NO˙, such as via enhanced peroxynitrite (ONOO^-^) formation [17]. Potentially, there are several determinants of this oxidatively-adapted pathway, including oxidatively inactivated sGC itself, oxidized NO˙, and PDE. The pharmacological challenge is how best to deploy potential therapeutic options that focus on these determinants under increased oxidative stress in a way that optimizes restoration of the function of this pathway.

Investigating the complexity of biological systems using combinatorial perturbations is a rational strategy for predicting function and phenotype [18], understanding network mechanisms [19–22], and identifying new and more promising therapeutic targets for human diseases [23, 24]. In theory, using a combination of drugs that can perturb different components of a system could be a more effective strategy than treating a disease with a single drug [25]. Indeed, the most complex diseases, such as cardiovascular diseases, cancer, diabetes mellitus, neurodegenerative diseases, and asthma, are multifactorial diseases. Systems-based interventions using multi-component drug combinations have been used increasingly to treat these complex diseases, although these approaches have largely been developed empirically in the clinical setting. The main purpose of model-based drug discovery is to revisit classical pharmacology logically in order to replace the one-gene, one-protein, and one-mechanism perspective with a systems-oriented paradigm to improve the therapeutic index of potential drugs targeting these complex diseases [26, 27].

Relevant principles have emerged from different studies of combination therapies that do not always yield predicted outcomes. For example, the combination of niacin (vitamin B_3_) with a statin [5-hydroxy-3-methylglutaryl-coenzyme A (HMG-CoA) reductase] leads to an incremental decrease in low-density lipoprotein (LDL) cholesterol concentration and an increase in high-density lipoprotein (HDL) cholesterol concentration [28]. Combinations of drugs that perturb five different targets in the HIV life cycle have turned AIDS from a lethal infection into a manageable chronic disease [29]. Another interesting combination is that of nitroglycerin and N-acetylcysteine (NAC), which can potentiate the effects of nitroglycerin in the treatment of acute myocardial ischemia [30]. The combination of β_2_-adrenergic receptor activators with muscarinic receptor blockers is useful for the treatment of chronic obstructive pulmonary disease [31].

Opposing and independent regulatory mechanisms within the NO˙-cGMP pathway determine the biological level of cGMP in the steady-state. Model-based approaches have facilitated our understanding of these regulatory mechanisms for cGMP formation [32–34]. Thus far, modeling has been used to study two distinct limbs of the NO˙-cGMP signaling pathway separately [33–39]; however, here, we will build this model as an integrated system that also includes oxidative inactivation. We then pose the question of whether a combination of two or three agents with orthogonal therapeutic actions (and toxicities), used at lower concentrations than when used alone, will enhance cGMP formation beyond that of single agents in the presence of oxidative stress.

In this study, we pursued this question using a dynamical model of the NO˙-cGMP signaling pathway in the presence of hydrogen peroxide. Impaired activation of NO˙-cGMP signaling has been observed in several cardiovascular disorders, including heart failure [40] and pulmonary arterial hypertension [41], due, in part, to excess oxidants. Current treatments for these disorders that involve this pathway includes nitrovasodilators and phosphodiesterase inhibitors. Thus, this theoretical approach, were it to demonstrate benefit, may offer initial strategies for optimal drug combinations for the treatment of these (and other) disorders in which NO˙-cGMP signaling is dysfunctional using approved agents.

Previous studies have used models of cell signaling networks to evaluate the action of drug pairs as compared with corresponding individual drugs [42]. Modeling drug action using ordinary differential equations could be challenging without sufficient information about the integrated network kinetics of drug action. To address this challenge, Araujo and colleagues investigated an interesting concept, perturbation simulation, on the epidermal growth factor receptor (EGFR) signaling network. They found that pairwise perturbations of reaction rates was more effective at restoring optimal function of the network than individual perturbation of corresponding reaction rates [43].

We expanded this approach to a practically remediable pathway and examined how hydrogen peroxide (H_2_O_2_)-induced oxidant stress affects the key reaction steps of the NO˙-cGMP signaling pathway to diminish cGMP levels, and then developed a combinatorial approach to perturb the oxidatively-impaired NO˙-cGMP signaling pathway and restore cGMP levels toward normal. In addition, in contrast to [43], we also examined the consequences of lesser degrees of inhibition in combination modeling to infer lower dose-dependent toxicity. Lastly, in contrast to [43], we performed cell-based experiments to validate the modeling strategy. To do so, all rate constants were perturbed individually, in pairs, or in trios by step-wise ten-fold changes in their values to their original values, which is analogous to concentration-dependent inhibition of a given reaction by a specific inhibitor. Our goal was to identify an optimal perturbation that augments the cGMP levels toward normal during oxidative stress.

Using a single perturbation, we found that the potential therapeutic targets, including the oxidation of sGC, NO˙ dissociation from sGC, and cGMP degradation by PDE, had a profound effect on enhancing cGMP accumulation as compared with other reaction steps. Using combined perturbations, we were able to identify an optimal triple perturbation that increases cGMP levels beyond that observed with the corresponding individual or paired perturbations that comprise it. Importantly, these theoretical findings were confirmed in cell-based experiments in which a combination of a nitric oxide donor (S-nitroso-N-penicillamine), an antioxidant (N-acetyl-L-cysteine), and a phosphodiesterase type 5-inhibitor (sildenafil) significantly improved the cyclic GMP output of the pathway in the setting of oxidant stress (hydrogen peroxide) in pulmonary artery vascular smooth muscle cells.

## Results

Elevated reactive oxygen species (ROS) can affect both the cGMP-synthetic limb [9, 10] and the cGMP-degradative limb [44] of the NO˙-cGMP signaling pathway. In order to assess the combinatorial effects of different pharmacological modulators on dynamical pathway behavior (as determined by cGMP output), we first modeled the dynamical behavior and then assessed the effects of optimal combinations of pharmacological inhibitors in cell-based assays using pulmonary artery vascular smooth muscle cells.

### Modeling the NO˙-cGMP Signaling Pathway

Once NO˙ is generated in source cells (endothelial cells) in the vasculature, it diffuses into vascular smooth muscle cells and binds to sGC, a ferrous iron hemoprotein receptor, to generate the NO˙-sGC complex. Either sGC alone or the NO˙-sGC complex, whose specific activity is ~200 times greater than sGC alone [45], can convert GTP to the second messenger molecule, cGMP, which is degraded by cyclic nucleotide PDE(s) to GMP. However, under oxidative stress conditions, sGC is also oxidized by H_2_O_2_ and thereby desensitized (Fig 1). The biological reactions comprising this system were modeled using ordinary differential equations and mass action kinetics involving 12 molecular species and 13 rate constants (S1 Table).

**Fig 2:**
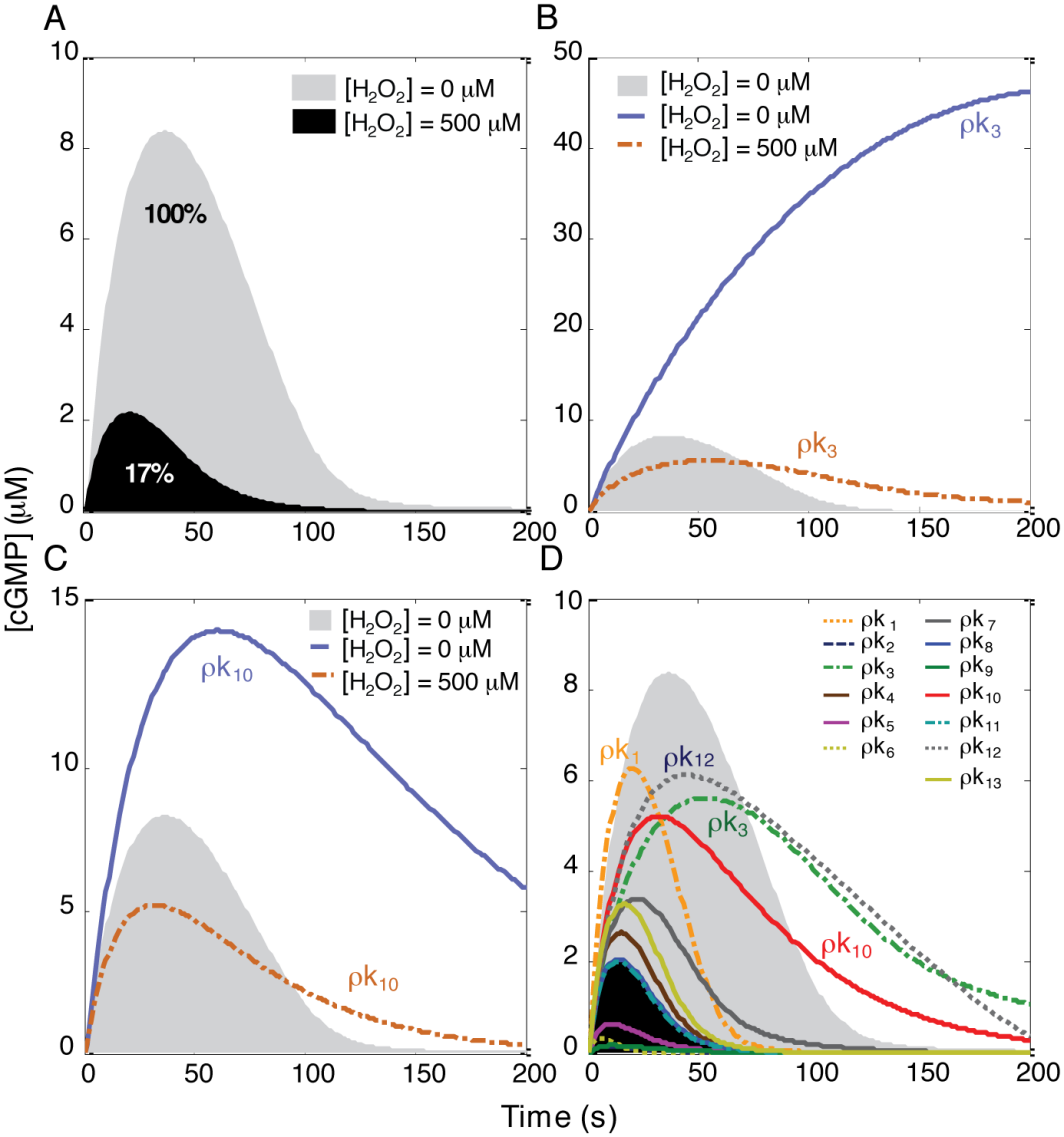
Monitoring cGMP levels after single perturbation of oxidatively impaired NO˙-cGMP pathway. (A) The effect of H_2_O_2_ on cGMP dynamics. H_2_O_2_ caused ~6-fold reduction in cGMP levels. (B and C) Reducing either k_3_ or k_10_ during oxidative stress was not an effective perturbation for restoring cGMP to the normal level. The value of k_3_ or k_10_ was reduced to 10% of their original values (S1 Table), with perturbation of k_3_ or k_10_ denoted as ρk_3_ and ρk_10_, respectively. While these perturbations can be effective strategies in enhancing cGMP levels in the absence of H_2_O_2_, they are not effective in restoring cGMP levels to normal in the presence of H_2_O_2_. (D) All single perturbations of the NO˙-cGMP pathway in the presence of H_2_O_2_. The cGMP levels were monitored in the absence of H_2_O_2_. Subsequently, in the presence of H_2_O_2_, the system was perturbed by reducing one of thirteen rate constants (k_1_‒k_13_) to 10% of their original values, denoted as ρks, and then the cGMP levels calculated. Note that ρk_1_, ρk_3_, ρk_10_, and ρk_12_ markedly increased the cGMP levels beyond other perturbations.

The simulation time intervals were selected to monitor cGMP dynamics between 0 and 200 seconds (based on the cGMP dynamics in S1A Fig for pulmonary artery vascular smooth muscle cells). Next, an oxidant (500 μM H_2_O_2_) was added to the system to alter the dynamics of all molecular species, including cGMP, as compared with control. One prior experimental study showed that both cGMP-degrading enzymes and sGC desensitization cooperatively accounted for the diverse patterns of cGMP responses to NO˙. Two different temporal dynamic signatures of cGMP were reported within platelets and astrocytes that have high and low levels of PDEs, respectively [32, 46, 47]. In our system under control conditions, the cGMP concentrations increased abruptly to a peak concentration within 40 seconds, and then decreased to baseline within 200 seconds (mirroring the experimental dynamics of S1A Fig). When the system was exposed to H_2_O_2_, the cGMP levels decreased by ~6-fold (Fig 2A). In this model, we proposed that H_2_O_2_ impairs activation of sGC and its generation of cGMP [48]; however, conflicting results have been reported by others [49].

### Relative Contributions of the Determinants of cGMP Synthesis and Degradation to the NO˙-cGMP Signaling Pathway

To restore cGMP generation to the normal level, there are several potential therapeutic targets, including oxidatively inactivated sGC, the NO˙-activated form of sGC, and PDE. Therapies for some diseases with PDE inhibitors have been successful at increasing cGMP levels in both cardiac and vascular tissues. However, to predict which one of these potential targets would be most effective at increasing cGMP levels, we perturbed either the synthetic limb (k_3_) or the degradative limb (k_10_) of the pathway in the absence or the presence of H_2_O_2_, and then evaluated cGMP dynamics. We found that targeting these two reaction steps can significantly increase the cGMP levels as compared with control if there is sufficient unoxidized sGC available. However, under significant oxidative stress, targeting these two reaction steps cannot be an effective strategy for restoring cGMP levels to normal (Fig 2B and 2C).

To evaluate more systematically the role of any given reaction in cGMP formation, we compared cGMP dynamics by reducing each of the thirteen rate constants to 10% of its original value (simulating significant reaction inhibition) in the presence of H_2_O_2_. We found that the cGMP levels were not restored toward control levels by decreasing k_1_ (oxidizing sGC), k_3_ (desensitizing sGC), or k_10_ (degrading cGMP) to 10% of their original values (Fig 2D). This observation suggested that under oxidative stress, targeting either the synthetic limb or the degradative limb of the pathway alone is not an effective approach for restoring cGMP to normal levels.

### Combined Perturbation of the Oxidized NO˙-cGMP Pathway

**Fig 3:**
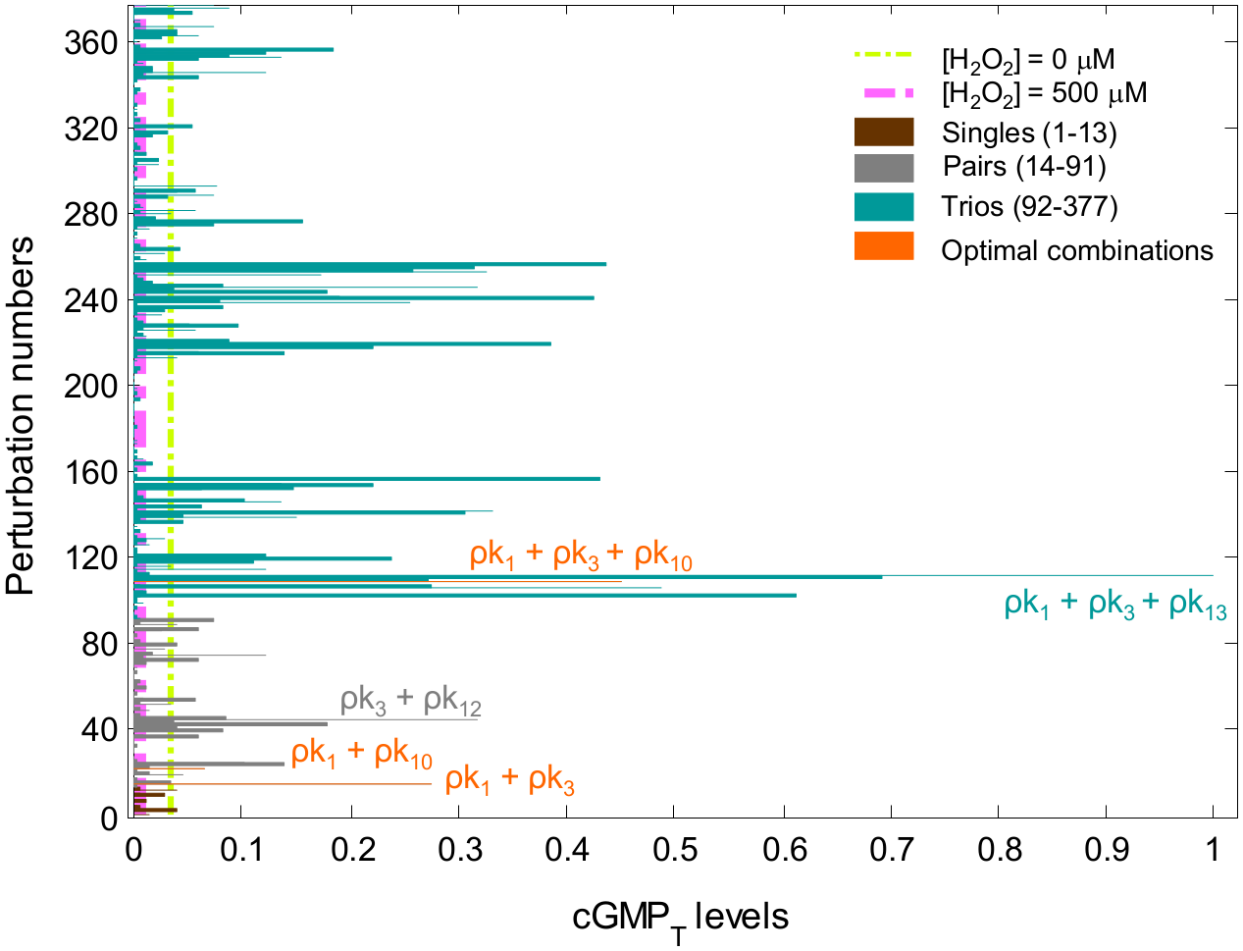
Relative cGMP_T_ levels after single, paired, and triple kinetic perturbations of the oxidatively impaired NO˙-cGMP pathway. The relative level of cGMP_T_ as a function of all possible single (13 brown bars), paired (78 gray bars), and triple (286 green bars) perturbations is shown. Values of the rate constants were reduced to 10% of their original values (S1 Table). Each bar shows the relative integrated cGMP levels over the period of simulation, or $cGMP_{\text{T}}=\int_{0}^{\text{T}}[cGMP](t)dt$. Note that some of the optimal perturbations are highlighted in this figure.

The relative involvement of both synthetic and degradative components of the NO˙-cGMP signaling pathway led us to propose that these components could exert autonomous effects on cGMP accumulation. This concept raised the possibility that combined perturbations may have more profound effects on cGMP levels than single perturbations. Addressing this concept, the NO˙-cGMP pathway was perturbed using all possible single, paired, or triple perturbations in the presence of H_2_O_2_, and then the time-integrated cGMP (cGMP_T_) levels were calculated (Fig 3). We found that the simultaneous perturbation (ρ) of several rate processes along with the perturbation of k_1_ (ρk_1_) yielded the highest cGMP_T_ levels relative to other perturbations. This finding suggested that targeting the primary driver of pathway dysfunction (ρk_1_) along with other potential therapeutic targets might be a better approach for increasing cGMP levels (even) beyond control levels under oxidative stress.

### Evaluation of the Effects of Combined Perturbations on cGMP Responses

**Fig 4:**
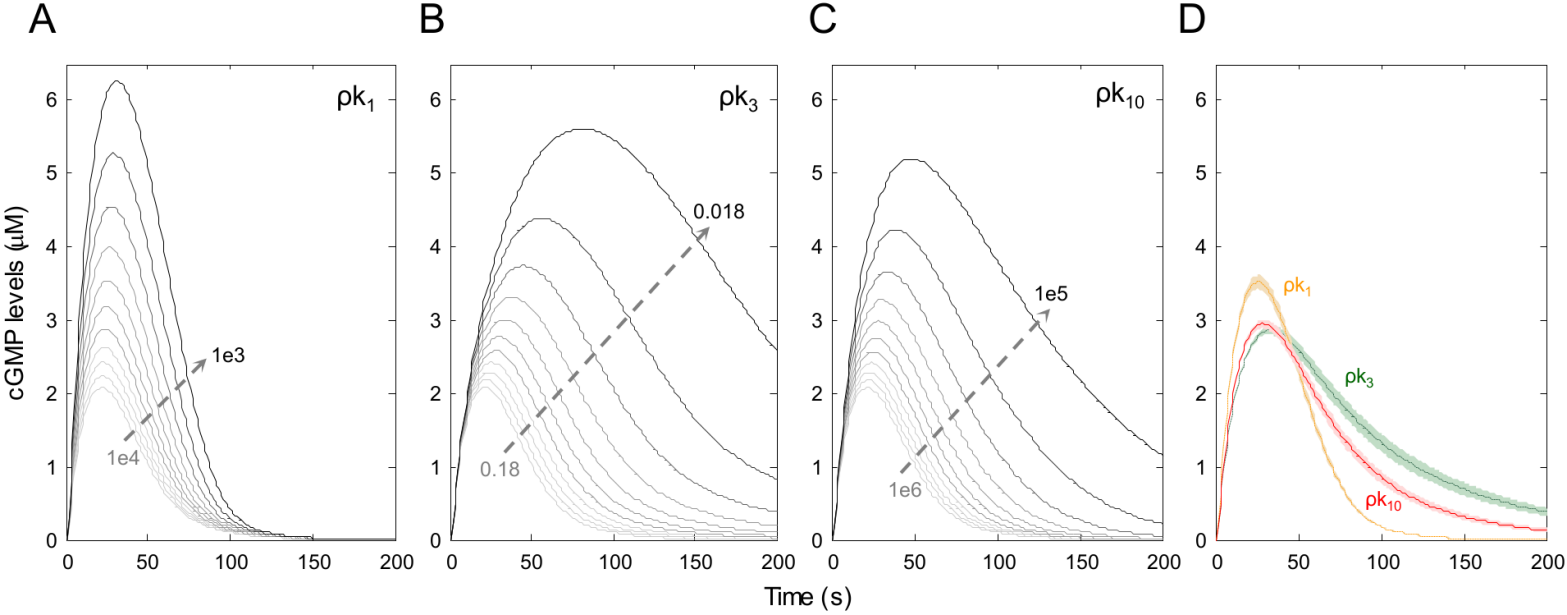
The effects of linear reduction of values for three individual rate constants on cGMP dynamics. Using the linear reduction of values for k_1_, k_3_, and k_10_, we created a vector of eleven different values for each of these rate constants. We next generated three linearly spaced vectors for each of the rate constants by fractionally reducing each decrementally. Using these vectors of rate constants, cGMP dynamics were calculated. The cGMP levels after serial reduction of (A) k_1_, (B) k_2_, and (C) k_3_. (D) The relative cGMP levels. Data are shown as mean (dashed lines) ± S.E.M (shaded lines) of 11 simulated replicates. Note: k_3_ was the most sensitive parameter in cGMP accumulation as compared with k_1_ and k_10_. In addition, the highest cGMP levels were achieved at ~40 seconds.

We next perturbed the proposed rate constants, including ρk_1_, ρk_3_, ρk_10_, or all possible combinations of these three rate constants, in the presence of H_2_O_2_. Modeling a fractional linear reduction of values for these rate constants, we created a vector of eleven different values for each wherein the maximum value for each rate constant was its original value (S1 Table) and the minimum value was 0.1, 0.3, or 0.5 of its original value for a rate constant in single, paired, or triple perturbations, respectively (lesser minimal values were used with greater combined perturbations to attempt to demonstrate efficacy at combined doses that might limit dose-dependent toxicities). Rate constants that were not varied under each set of modeling conditions were maintained at their full values. The cGMP dynamics was then calculated using the range of rate constants (Fig 4). These results suggested that under oxidative stress, decreasing dissociation of NO˙ from the NO˙-sGC complex (ρk_3_) is the most sensitive reaction step for increasing cGMP levels as compared with the use of an anti-oxidant (ρk_1_) and PDE inhibitor (ρk_10_). Furthermore, perturbation of k_1_ (ρk_1_) is the best strategy by which to increase cGMP levels beyond perturbation of either k_3_ (ρk_3_) or k_10_ (ρk_10_).

**Fig 5:**
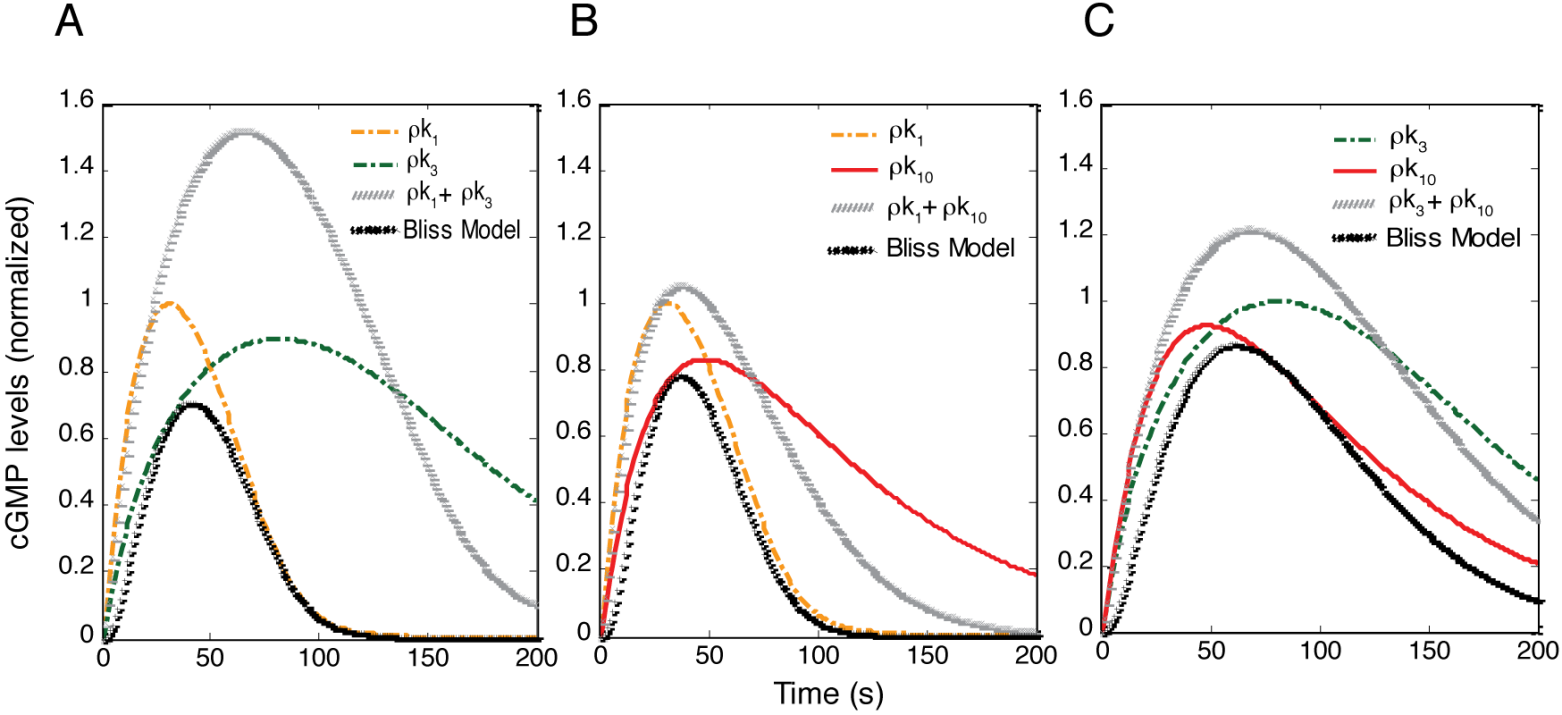
Pairwise perturbation of optimal rate constants increased cGMP levels beyond that predicted by the Bliss model. Comparison of cGMP levels after individual and pairwise perturbation of (A) k_1_ + k_3_, (B) k_1_ + k_10_, and (C) k_3_ + k_10_ with the Bliss model [generated using the perturbation of corresponding individual rate constants and applying eq (15) in the Supplement]. Single perturbations were used to predict paired perturbation signatures. The simulated combination produced cGMP levels that were greater than those predicted by the Bliss model.

**Fig 6:**
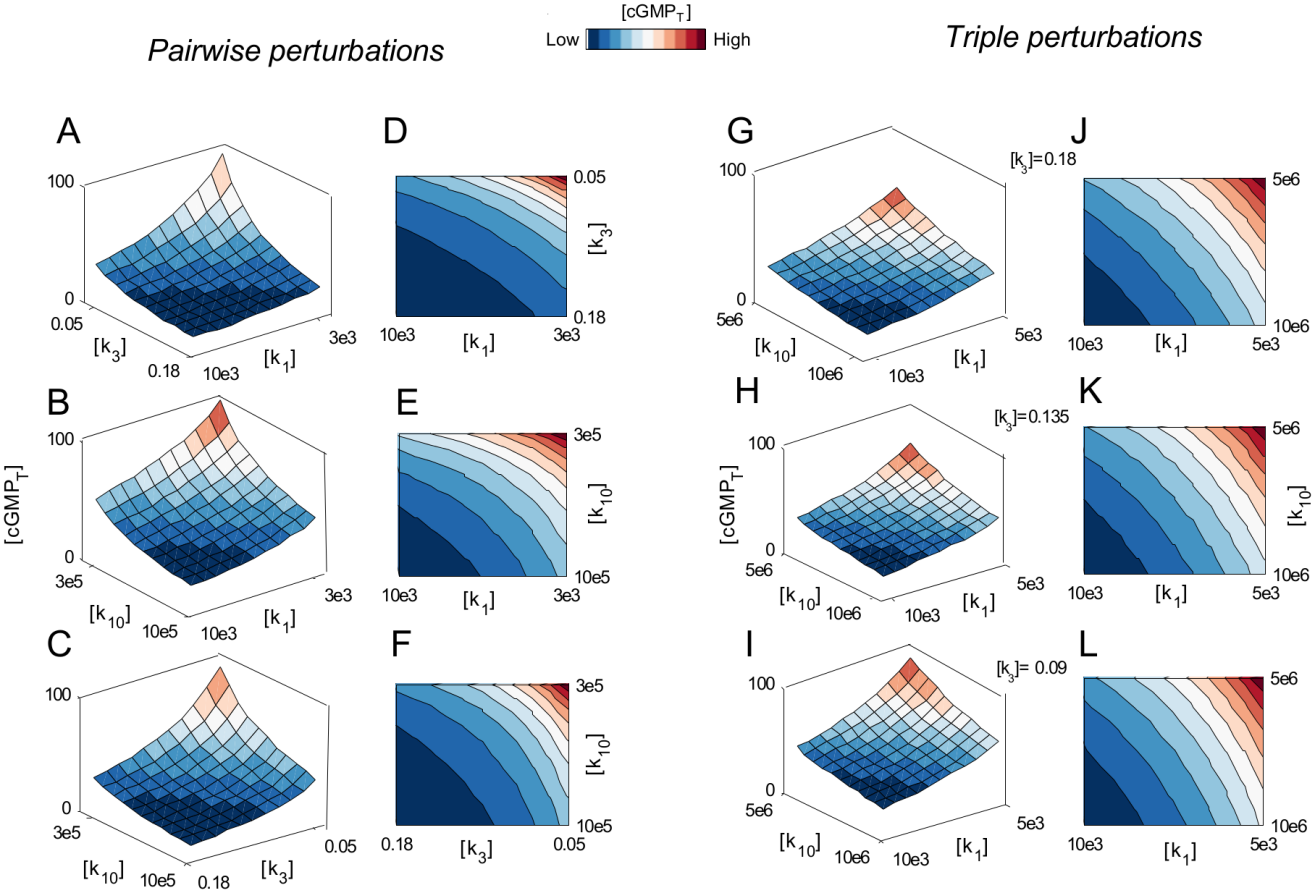
Pairwise or triple perturbations of three agents additively enhance the cGMP_T_ levels. (A-C) The cGMP_T_ dose matrix-responses to all possible combined perturbations of three rate constants. We iteratively reduced the rate constants by fractional decrements with *ρ* defined as the (normalized) perturbation ratio with values either 0.3 or 0.5 for pairwise or triple perturbations, respectively. For pairwise perturbations, two vectors of rate constants were combined in 11×11 matrices in which the value of each rate constant was fractionally reduced by linear decrements along each axis. The combined effects of (A) k_1_ plus k_3_, (B) k_1_ plus k_10_, and (C) k_3_ plus k_10_ on cGMP_T_ levels. (D-E) The contour plots illustrate that the pairwise perturbations exerted additive effects on cGMP_T_ levels. (G-I) The cGMP dose matrix responses to the triple perturbation of k_1_, k_3_, and k_10_. Three vectors of rate constants were combined in 11×11×11 matrices in which the value of each rate constant was linearly reduced along each axis. (J-L) The contour plots reveal additive augmentation of cGMP_T_ levels.

Subsequently, we used the Bliss model [50] (which, based on probability theory, assumes two inhibitors work through independent mechanisms of action, and assumes that the two inhibitors do not interfere or compete with each other) to evaluate the power of paired perturbations. Under oxidative stress, optimal parameters were perturbed either individually or in pairs in order to compare the effects of perturbations on cGMP levels. The effects of single perturbations on cGMP levels were used to calculate the Bliss model, as indicated by eq (15). As depicted in Fig 5, paired perturbations of optimized single parameters increased cGMP levels beyond the Bliss model predictions. To assess whether these differences are additive or non-additive, we used an isobologram analysis. We examined the combined effects on cGMP_T_ when two or three rate constants were perturbed simultaneously. The isobologram (contour plot) [51–56] was used to quantify the combined effects (Fig 6), wherein we observed that combined perturbations act additively to increase cGMP levels in this system.

### cGMP Dynamics in the Absence and Presence of H_2_O_2_

We studied cGMP dynamics using human embryonic kidney (HEK) 293 cells and human pulmonary artery vascular smooth muscle (PAVSM) cells. PAVSM contain abundant PDE5 compared with HEK293 cells (35), thereby ensuring that both the cGMP-synthetic and degradative limbs determine the cGMP levels (S1 Fig). Thus, in PAVSM, the rapid accumulation of cGMP is followed by its equally rapid reduction (S1A Fig). In HEK293 cells, which contain lower amounts of PDE5 (35) as compared with PAVSM cells, the cGMP-synthetic limb of the pathway primarily determines the cGMP levels (S1B Fig). The cells were pretreated with either H_2_O_2_ or buffer for 30 minutes. Time points were selected to capture cGMP dynamics. When the HEK293 cells were exposed to H_2_O_2_ at 500 μM, we found that NO˙-stimulated cGMP production was significantly reduced as a function of time (S1B Fig). This result suggested that H_2_O_2_ blocked the cGMP-synthetic limb of the pathway, which plays a predominant role in determining the cGMP levels in HEK293 cells (as confirmed by the absence of a biphasic response in cGMP dynamics compared with the PAVSM cells in S1 Fig).

### cGMP Dynamics in the Presence of Pharmacological Modulators of the cGMP Pathway

**Fig 7:**
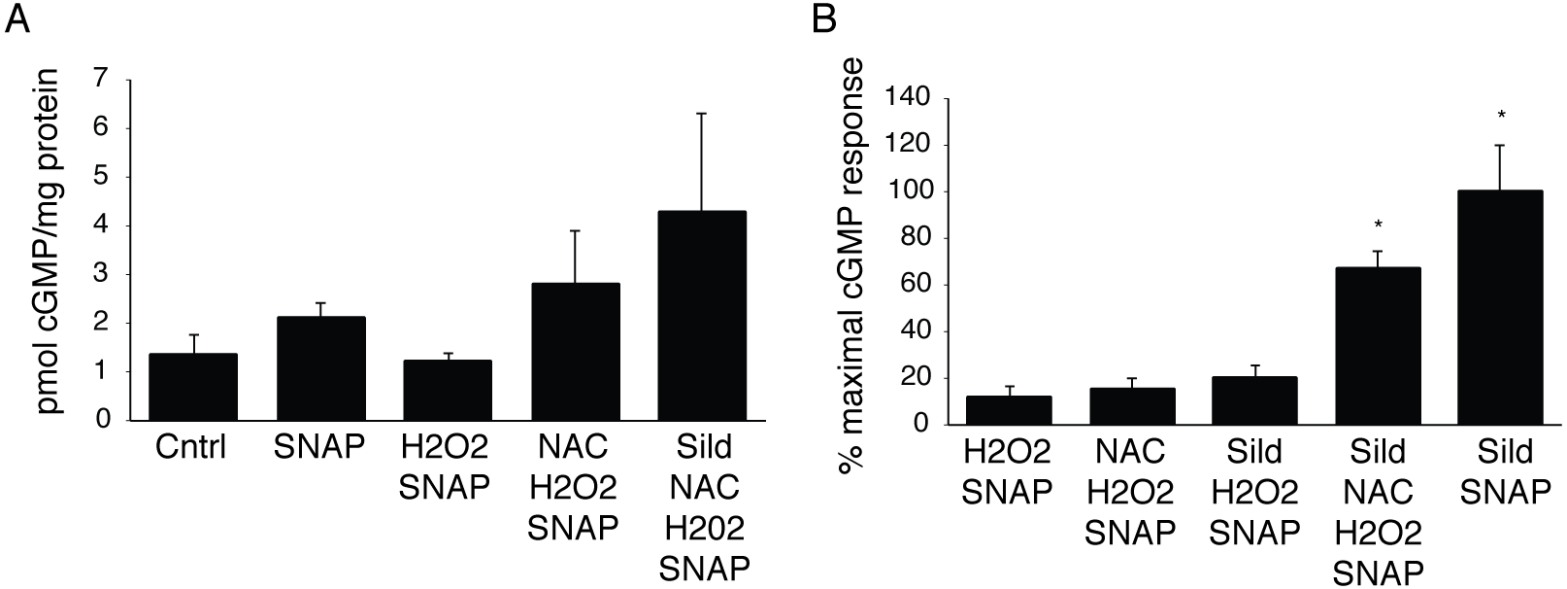
Increases in cGMP in PAVSM cells in the presence and absence of pharmacological agents that affect the cGMP pathway. In (A) and (B), PAVSM cells were pre-incubated for 90 min with buffer, 10 mM NAC (N-acetylcysteine), or 100 nM sildenafil (sild), followed by 30 min incubation with 500 μM hydrogen peroxide. Cells were then incubated with 100 μM SNAP for 10 min prior to harvesting for cGMP determination. Panel (A) shows a representative experiment, with three biological replicates under each condition. Shown in (B) are the averages of 3–4 separate experiments with each condition tested in duplicate or triplicate. Sildenafil, NAC, H_2_O_2_, plus SNAP condition was not significantly different from the sildenafil plus SNAP condition; these two conditions (* indicates p<0.05 by ANOVA followed by a Newman-Keuls test) were significantly different from all other conditions.

In order to determine the validity of the combinatorial modeling described above, we measured cGMP in PAVSM cells treated with various combinations of agents that act on different steps in the pathway of Fig 1. Agents were chosen because they have been used in human studies, and because they affect each of the limbs of the pathway in Fig 1. As shown in Fig 7A, we first showed that the addition of a NO^.^-donor, S-acetyl-N-penicillamine (SNAP), increased the cGMP produced by 58% over vehicle-treated control cells; hydrogen peroxide treatment, however, abrogated this increase. When cells were treated with hydrogen peroxide and the reducing agent, N-acetyl-L-cysteine (NAC), cGMP levels increased to ~2-fold above control. The addition of sildenafil, a PDE5 inhibitor (the primary PDE isoform found in PAVSM responsible for cGMP degradation), to SNAP and NAC in the presence of hydrogen peroxide further increased cGMP levels to ~3.2-fold above vehicle-treated control cells. With these baseline measurements, we next explored key comparative combinations of agents that mimicked the optimal modeled combinations, as shown in Fig 7B. Here, cGMP responses are reported as the % of the maximal response (to sildenafil and SNAP in the absence of hydrogen peroxide) owing to variation from experiment to experiment. We observed that the combinations of NAC and SNAP, sildenafil and SNAP, and sildenafil, NAC, and SNAP each increased cGMP in the presence of hydrogen peroxide, and that the relative magnitude of the increases was consistent with the modeled data in Fig 3. The use of NAC inhibits reactions 1 (and possibly 13), the use of SNAP ‘inhibits’ reaction 3 indirectly by driving reaction 2, and the use of sildenafil inhibits reaction 10 as a competitive inhibitor of PDE5 and indirectly inhibits reaction 12 by limiting the formation of the catalytic complex and hence substrate turn-over. Thus, as in Fig 3, the magnitude of increase in cGMP was of the following order: inhibition of reactions 1 + 3 < inhibition of reactions 3 + 10 (or 12) < inhibition of reactions 1 + 3 + 10, which is similar to the experimental reaction order we observed in the data of Fig 7.

## Discussion

Impaired activation of NO˙-cGMP signaling pathway has been observed in cardiovascular disorders and other common disease states. There are multiple enzymatic determinants of cGMP production in this pathway, including sGC itself, the oxidatively inactivated form of sGC, the NO˙-activated form of sGC, enzymatic sources of NO˙, and PDE. Therapies for these disorders with PDE inhibitors have been successful at enhancing cGMP levels in cardiac and vascular tissue with attendant improvement in lusitropy and vasodilation, respectively. However, PDE is only one of the enzymatic determinants of cGMP formation. In this systems-level approach, we used all possible single, paired, or triple perturbations to propose a combined perturbation that was more effective in cGMP accumulation than any single perturbation. The optimal number of the perturbations was three owing to there being only three key processes that determine independently the cGMP levels, i.e., cGMP synthesis, cGMP degradation, and oxidative inactivation of sGC. By having this modeled information, one can improve experimental design, curb cost, and save time in performing the experiments necessary for gaining useful results. Alternatively, one could randomly target any given component of this pathway either individually or in combination with other components of the pathway. Yet another approach is the maximal damage targeting strategy [57], theoretically a better approach relative to the random targeting of a pathway. However, using either the random targeting or the maximal damage targeting approach, we might overlook the optimal perturbations among many combinations that may never have been tested.

PDEs are essential enzymes within normal cells that degrade the phosphodiester bond in the second messenger molecules cAMP and cGMP. PDEs are, therefore, important regulators of signal transduction mediated by these second messenger molecules. As with many drugs that affect molecular pathways involved in (many) different signaling pathways, the side effects of PDE inhibitors are dose-dependent [58]; thus, to reduce the dose of a PDE inhibitor and then combine it with other potential drugs that have non-overlapping mechanisms of action and toxicities may significantly improve the overall therapeutic index of the treatment strategy.

One of the rationales for using combination therapy is to block redundant pathways that exist extensively within the molecular networks whose functions are modified in human diseases. To overlook this network property may limit the potential for reformulating existing drugs that can be used in combination with higher efficacy and fewer toxicities. Our results show how the combined perturbations of the NO˙-cGMP signaling pathway represent a useful strategy for increasing cGMP levels. A model-based analysis suggests that the combinatorial perturbation of biological networks is a promising approach by which to identify drug combinations with higher efficacy and perhaps lower toxicity (rational polypharmacy) [42]. Further work on other specific pathways will be required to validate the general approach.

## Materials and Methods

### cGMP Measurements

Enzyme immunoassay (EIA): human pulmonary artery vascular smooth muscle (PAVSM) cells and growth media were obtained from Lonza Inc. (Walkersville, MD., USA). Confluent cells were pre-treated with phenol-red free DMEM (supplemented with 10% fetal calf serum) in the presence of absence of 10 mM N-acetyl-L-cysteine (NAC), a thiol reducing agent and antioxidant, to reverse mildly oxidized critical sulfhydryl groups in sGC [14] and, possibly, to prevent the oxidation of NO to NO_x_; and/or 100 nM sildenafil, a PDE5 inhibitor, for 90 min followed by 500 μM H_2_O_2_ for 30 min. Cells were next treated with either phosphate-buffered saline or 100 μM S-nitroso-N-acetylpenicillamine (SNAP), a NO^.^-donor, for 10 min. PAVSM cells were rinsed in ice-cold phosphate buffered saline and then solubilized in ice-cold 6% trichloroacetic acid. Samples were stored at -80° until the day of the assay. Samples were processed and cGMP and protein were measured as previously described [14]. cGMP formation was measured by immunoassay according to the cGMP Assay (Cayman Chemical Co., Ann Arbor, MI). H_2_O_2_, trichloroacetic acid, NAC, and sildenafil were purchased from Sigma-Aldrich (St. Louis, MO). Phenol-red-free DMEM was obtained from Gibco, Life Technologies, Grand Island, NY and fetal calf serum was from Atlanta biologicals, Flowery Branch, GA.

### Constructing the Model

Assuming mass-action kinetics, the reaction scheme (Fig 2, S1 Table) was deconstructed into 12 ordinary differential equations (ODEs):

$$d\;[H_{2}O_{2}]/dt\;=\;-k_{1}\;[\;H_{2}O_{2}]\;*\;[sGC]$$ (1)

$$\begin{array}{l} d\;[sGC]/dt\;=\;-\;k_{1}\;[H_{2}O_{2}]\;*\;[sGC]-k_{2}\;[NO^{.}]*\;[sGC]\;+k_{3}\;[NO^{.}-sGC]-k_{4}\;[\;sGC]\;*\;[GTP]\;\\ \;+\;(k_{8}\;+k_{5})\;[sGC-GTP] \end{array}$$ (2)

$$d\;[sGC-H_{2}O_{2}]/dt\;=\;k_{1}\;[\;H_{2}O_{2}]\;*\;[sGC]$$ (3)

$$d\;[NO^{.}]/dt\;=\;-k_{2}\;[\;NO^{.}]\;*\;[sGC]+k_{3}\;[NO^{.}-sGC]-k_{13}[NO^{.}]$$ (4)

$$\begin{array}{l} d\;[NO^{.}-sGC]/dt\;=\;k_{2}\;[\;NO^{.}]\;*\;[sGC]-k_{3}\;[NO^{.}-sGC]-k_{6}\;[GTP]*\;[NO^{.}-sGC]\;+\\ \;(k_{9}+k_{7})\;[NO^{.}-sGC-GTP] \end{array}$$ (5)

$$\begin{array}{l} d\;[GTP]/dt\;=\;-k_{4}\;[\;GTP]\;*\;[sGC]+k_{5}\;[sGC-GTP]-k_{6}\;[GTP]*\;[NO^{.}-sGC]\;\\ \;+\;k_{7}\;[NO^{.}-sGC-GTP]\; \end{array}$$ (6)

$$d\;[sGC-GTP]/dt\;=\;k_{4}\;[\;GTP]\;*\;[sGC]-(k_{5}+k_{8})\;[sGC-GTP]$$ (7)

$$d\;[NO^{.}-sGC-GTP]/dt\;=\;k_{6}\;[\;GTP]\;*\;[NO^{.}-sGC]-(k_{7}+k_{9})\;[NO^{.}-sGC-GTP]$$ (8)

$$\begin{array}{l} d\;[cGMP]/dt\;=\;k_{8}\;[\;sGC-GTP]\;+k_{9}\;[NO^{.}-sGC-GTP]-k_{10}[cGMP]*[PDE]\\ \;+k_{11}[cGMP-PDE] \end{array}$$ (9)

$$d\;[PDE]/dt\;=\;-k_{10}\;[\;cGMP]\;*[PDE]+(k_{11}\;+k_{12})\;[cGMP-PDE]$$ (10)

$$d\;[cGMP-PDE]/dt\;=\;k_{10}\;[\;cGMP]\;*[PDE]-(k_{11}\;+k_{12})\;[cGMP-PDE]$$ (11)

$$d\;[GMP]/dt\;=\;k_{12}\;[cGMP-PDE]$$ (12)

to simulate the dynamics of the molecular species within the NO˙-cGMP signaling pathway. The ODEs were solved using a numerical ODE solver (ode15s). All mathematical modeling and simulations were performed using the SimBiology toolbox in MatLab (Version 8, 2012b, MathWorks, Natick, MA). The parameter values for this model include 13 rate constants and 12 initial concentrations (S1 Table), which were chosen or estimated from the literature, as indicated in the Table. The system dynamics were assessed in the absence or presence of H_2_O_2_.

### Model Perturbations

In the presence of H_2_O_2_ (500 μM), the NO˙-cGMP pathway was perturbed by assuming the presence of an effective inhibitor of a given reaction(s) sufficient to impair the reaction kinetics (ρk = 0.1k). We perturbed all possible individual (13), pairs (78), or trios (286) of reactions in the model. The total number of perturbations (up to triple perturbations) was computed by inserting the total number of rate constants (q = 13) and the maximum number of perturbations (p = 3) into following equation:

Cqp=∑i=1pq!/[(q−i)! × i! (13)

Thus, the total possible number of perturbations (for triple perturbations) is _13_*C*_3_ = 377. To assess the relative role of each perturbation, cGMP dynamics were illustrated (Fig 3).

The time-integrated cumulative cGMP (cGMP_T_) level is defined as:

$$cGMP_{T}\;=\;\int_{0}^{T}[cGMP]\;(t)\;dt\;,\;T=\;200\text{sec}$$ (14)

#### Comparing the simulated pairwise perturbations with the Bliss model

We next used the Bliss model [50] to predict the effects of paired perturbations using the product effect of corresponding single perturbations. The Bliss model was used to evaluate whether combined perturbations enhance cGMP levels beyond corresponding single perturbations. The combined perturbation of two rate constants (ρk) is computed as the product of the individual perturbations of the two rate constants, ρk_i_ and ρk_j_.

$$\rho k=\;\rho k_{i}\;*\;\rho k_{j}$$ (15)

### Evaluating the Combined Perturbations Using Contour and Dose Matrix Plots

Perturbation of k_1_, k_3_, or k_10_ alone can induce a dose-dependent cGMP_T_ response in the presence of H_2_O_2_. We varied the rate constants by fractional linear decrements.

To depict the matrix response of cGMP_T_, two vectors of rate constant values were combined in 11×11 matrices where the value of each rate constant is depicted along each axis (Fig 6). The contour plots were used to evaluate additive and non-additive effects. Thus, the combined actions were either additive (Fig 6) or synergistic if the isobole is a straight line or a convex line, respectively. Likewise, 11×11×11 matrix of all model parameters was constructed for triple perturbations and three-dimensional contour plots analyzed accordingly.

## Supporting Information

S1 Text Text and references. (DOCX)

S1 Table The molecular species, initial concentrations, parameters and constant rates used to create the model. (DOCX)

S1 Fig The cGMP dynamics in the absence and the presence of H_2_O_2_. (TIF)
