# Supplementary material for: Systems Pharmacology and Rational Polypharmacy: Nitric Oxide−Cyclic GMP Signaling Pathway as an Illustrative Example and Derivation of the General Case
Source: PLoS Comput Biol. 2016 Mar 17;12(3):e1004822. doi: 10.1371/journal.pcbi.1004822 (PMC4795786; doi:10.1371/journal.pcbi.1004822)
Supplement: S1 Text — (DOCX) [file pcbi.1004822.s001.docx]

S1. Supplemental Text

Mathematical Modeling of NO˙-cGMP Signaling Pathway

The modeling approach has been used to study two distinct limbs of the NO˙-cGMP signaling pathway separately (1-7). In contrast, we built this model as an integrated system. By considering this more holistic notion, we required the use of both previously published and estimated parameters to build the model to monitor model performance. In this model, we used H_2_O_2_ at 500 µM to oxidize sGC and reduce cGMP levels significantly below control.

The steady-state concentrations for sGC, NO˙, GTP, and PDE were obtained from the literature. The steady-state sGC levels within arterioles was 100 nM (8). This concentration for nitric oxide was 0.24 µM; however, a fully activated macrophage can produce 1 µM nitric oxide per minute (9). Importantly this is a peak concentration in that the NO˙ concentration is diluted by diffusing into vascular smooth muscle cells. The physiological concentrations of cGMP are 0.1–10 µM (10). The calculated intracellular PDE5 concentration in heart was 13 ± 1.5 nM, which is ~12-fold lower than that measured in lung, 158 ± 50 nM (11). PDE5 concentration was also reported at 188 ± 6 nM (12). It is known that nitric oxide is scavenged within 0.1 second (13) or a few seconds (9) through reaction with oxyhemoglobin. Kinetic studies of soluble guanylyl cyclase complexed with nitric oxide demonstrate that NO˙ dissociation in the presence of the substrate GTP and Mg^2+^ is as much as 50 times faster than in their absence. In the presence of those two species, the dissociation rate constant is (0.04 ± 0.01 s^−1^) at 20 °C, which is by far the fastest NO˙ dissociation rate constant reported for a ferrous heme protein (14). In the presence of the substrate GTP, the NO˙-heme off-rate is also quite rapid (0.18 ± 0.01s^-1^) (15).

cGMP measurements in HEK cells

Intracellular cGMP in live HEK293 cells was monitored using the pGloSensor-42F detection kit purchased from Promega (Madison, WI, USA) using the directions provided and a GloMax 96-well luminometer from Promega.

**References**

1. Batchelor AM, Bartus K, Reynell C, Constantinou S, Halvey EJ, Held KF, et al. Exquisite sensitivity to subsecond, picomolar nitric oxide transients conferred on cells by guanylyl cyclase-coupled receptors. Proc Natl Acad Sci U S A. 2010 Dec 21;107(51):22060-5.

2. Condorelli P, George SC. In vivo control of soluble guanylate cyclase activation by nitric oxide: a kinetic analysis. Biophys J. 2001 May;80(5):2110-9.

3. Garthwaite J. Dynamics of cellular NO-cGMP signaling. Front Biosci. 2005;10:1868-80.

4. Halvey EJ, Vernon J, Roy B, Garthwaite J. Mechanisms of activity-dependent plasticity in cellular nitric oxide-cGMP signaling. J Biol Chem. 2009 Sep 18;284(38):25630-41.

5. Held KF, Dostmann WR. Sub-nanomolar sensitivity of nitric oxide mediated regulation of cGMP and vasomotor reactivity in vascular smooth muscle. Front Pharmacol. 2012;3:130.

6. Roy B, Garthwaite J. Nitric oxide activation of guanylyl cyclase in cells revisited. Proc Natl Acad Sci U S A. 2006 Aug 8;103(32):12185-90.

7. Yang J, Clark JW, Bryan RM, Robertson CS. Mathematical modeling of the nitric oxide/cGMP pathway in the vascular smooth muscle cell. Am J Physiol Heart Circ Physiol. 2005 Aug;289(2):H886-97.

8. Tsoukias NM, Kavdia M, Popel AS. A theoretical model of nitric oxide transport in arterioles: frequency- vs. amplitude-dependent control of cGMP formation. Am J Physiol Heart Circ Physiol. 2004 Mar;286(3):H1043-56.

9. Beckman JS, Koppenol WH. Nitric oxide, superoxide, and peroxynitrite: the good, the bad, and ugly. Am J Physiol. 1996 Nov;271(5 Pt 1):C1424-37.

10. Trivedi B, Kramer RH. Real-time patch-cram detection of intracellular cGMP reveals long-term suppression of responses to NO and muscarinic agonists. Neuron. 1998 Oct;21(4):895-906.

11. Corbin JD, Beasley A, Blount MA, Francis SH. High lung PDE5: a strong basis for treating pulmonary hypertension with PDE5 inhibitors. Biochem Biophys Res Commun. 2005 Sep 2;334(3):930-8.

12. Corbin JD, Kotera J, Gopal VK, Cote RH, Francis SH. Regulation of cyclic nucleotide levels by sequestration. In: Bradshaw RA, Dennis EA, editors. Handbook of Cell Signaling. San Diego: Academic Press; 2003. p. 465-70.

13. Kelm M, Schrader J. Nitric oxide release from the isolated guinea pig heart. Eur J Pharmacol. 1988 Oct 18;155(3):317-21.

14. Kharitonov VG, Russwurm M, Magde D, Sharma VS, Koesling D. Dissociation of nitric oxide from soluble guanylate cyclase. Biochem Biophys Res Commun. 1997 Oct 9;239(1):284-6.

15. Cary SP, Winger JA, Marletta MA. Tonic and acute nitric oxide signaling through soluble guanylate cyclase is mediated by nonheme nitric oxide, ATP, and GTP. Proc Natl Acad Sci U S A. 2005 Sep 13;102(37):13064-9.

16. Stone JR, Marletta MA. Spectral and kinetic studies on the activation of soluble guanylate cyclase by nitric oxide. Biochemistry. 1996 Jan 30;35(4):1093-9.

17. Traut TW. Physiological concentrations of purines and pyrimidines. Mol Cell Biochem. 1994 Nov 9;140(1):1-22.
