# Supplementary material for: Systems Pharmacology and Rational Polypharmacy: Nitric Oxide−Cyclic GMP Signaling Pathway as an Illustrative Example and Derivation of the General Case
Source: PLoS Comput Biol. 2016 Mar 17;12(3):e1004822. doi: 10.1371/journal.pcbi.1004822 (PMC4795786; doi:10.1371/journal.pcbi.1004822)
Supplement: S1 Table — (DOCX) [file pcbi.1004822.s002.docx]

| **Table S1.** The molecular species, initial concentrations, parameters and constant rates used to create the model | | | | | | |
| --- | --- | --- | --- | --- | --- | --- |
| **Bio-molecules** | **Concentrations (M)** | **References** | **Parameters** | **Rates (Unit)** | **Reactions** | **References** |
| [H_2_O_2_] | 0.0 or 5.0E-04 |  | k_1_ | 1.0E+04 (M^-1^S^-1^) | H_2_O_2_ + sGC → sGC-H_2_O_2_ |  |
| [sGC] | 1.0E-07 | (8) | k_2_ | 5.0E+06 (M^-1^S^-1^) | NO˙ + sGC → NO˙-sGC | (16) |
| [sGC-H_2_O_2_] | 0 |  | k_3_ | 0.18 (S^-1^) | NO˙ + sGC ← NO˙-sGC | (15) |
| [NO˙] | 2.4E-07 | (9) | k_4_ | 1.0E+06 (M^-1^S^-1^) | sGC + GTP → sGC-GTP |  |
| [NO˙-sGC] | 0 |  | k_5_ | 180.0 (S^-1^) | sGC + GTP ← sGC-GTP |  |
| [GTP] | 3.05E-04 | (17) | k_6_ | 1.0E+06 (M^-1^S^-1^) | NO˙-sGC + GTP → NO˙-sGC-GTP |  |
| [sGC-GTP] | 0 |  | k_7_ | 43.0 (S^-1^) | NO˙-sGC + GTP ← NO˙-sGC-GTP |  |
| [NO˙-sGC-GTP] | 0 |  | k_8_ | 0.15 (S^-1^) | sGC-GTP → sGC + cGMP |  |
| [cGMP] | 0 | (10) | k_9_ | 28.7 (S^-1^) | NO˙-sGC-GTP → NO˙-sGC + cGMP |  |
| [PDE] | 1.5E-07 | (11) | k_10_ | 1.0E+06 (M^-1^S^-1^) | cGMP + PDE → cGMP-PDE |  |
| [cGMP-PDE] | 0 |  | k_11_ | 0.13 (S^-1^) | cGMP + PDE ← cGMP-PDE |  |
| [GMP] | 0 |  | k_12_ | 2.2 (S^-1^) | cGMP-PDE → PDE + GMP |  |
| [NO_x_] | 0 |  | k_13_ | 1 (S^-1^) | NO˙ → NO_x_ | (13) |

Abbreviations: cGMP, guanosine 3', 5'-monophosphate; GMP, guanosine-5'-monophosphate; GTP, guanosine-5'-triphosphate; H_2_O_2_, hydrogen peroxide; M, molar; NO˙, nitric oxide; NO_x_, oxidized (inactive nitrogen oxides); PDE, phosphodiesterase; sGC, soluble guanylyl cyclase
