# Supplementary figures and images for: Systems Pharmacology and Rational Polypharmacy: Nitric Oxide−Cyclic GMP Signaling Pathway as an Illustrative Example and Derivation of the General Case
Source: PLoS Comput Biol. 2016 Mar 17;12(3):e1004822. doi: 10.1371/journal.pcbi.1004822 (PMC4795786; doi:10.1371/journal.pcbi.1004822)

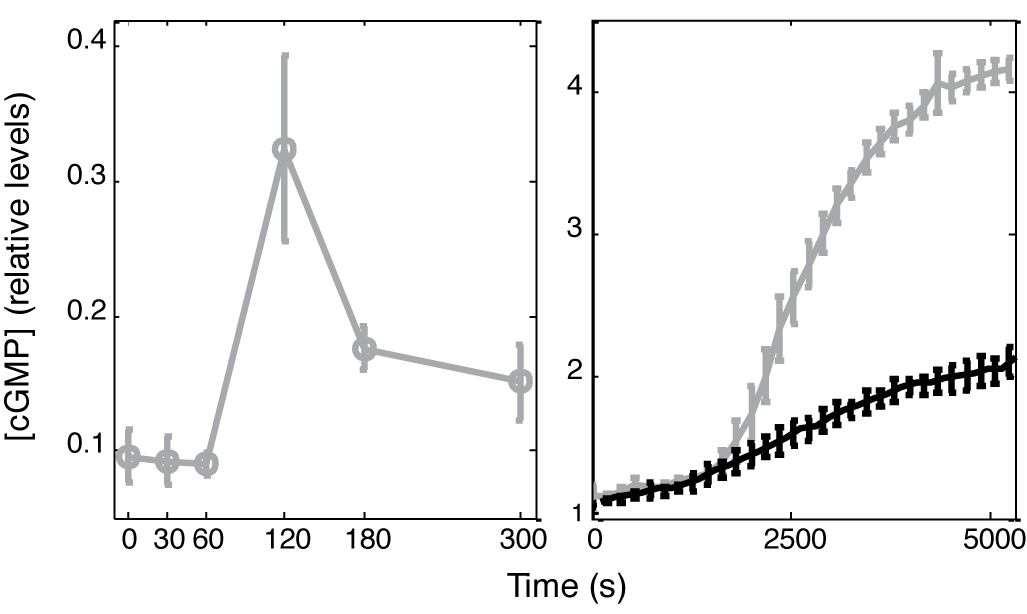

Supplement: S1 Fig — (TIF) [file pcbi.1004822.s003.tif]
